# The impacts of high refractive errors on self‐reported visual function and visual concern

**DOI:** 10.1111/aos.70120

**Published:** 2026-03-07

**Authors:** Jens Riis Møller, Arevak Saruhanian, Jens Rovelt, Josefine Freiberg, Marcel Reimann, Jeppe Nygård Samuelsen, Christina Eckmann‐Hansen, Miriam Kolko, Nina Jacobsen, FOREVER Consortium

**Affiliations:** ^1^ Department of Drug Design and Pharmacology University of Copenhagen Copenhagen Denmark; ^2^ Technical University of Denmark Kongens Lyngby Denmark; ^3^ Department of Ophthalmology, Copenhagen University Hospital, Rigshospitalet Glostrup Denmark; ^4^ Department of Ophthalmology, Aalborg University Hospital Aalborg Denmark; ^5^ Dine Øjenlæger (your Eye Doctors) Rungsted Denmark; ^6^ Frederiksborggade Øjenklinik Copenhagen Denmark

**Keywords:** hypermetropia, myopia, questionnaire data, refractive error, self‐perceived visual function, self‐reported eye health

## Abstract

**Purpose:**

To examine the association between refractive errors and self‐reported visual function and visual concern, considering self‐perceived present eyesight, concerns about vision, accomplishing less and limitations in daily activities in an eye‐healthy cohort.

**Methods:**

A cross‐sectional design was used, drawing upon data from a Danish eye and vision cohort, Project FOREVER. Data included refractive measurements and questionnaire responses from participants aged 18 years and above. Logistic regression was applied to investigate the association between self‐reported visual function, visual concern and refractive measurements. Refraction was grouped based on spherical equivalent values. Participants were defined as eye‐healthy without the presence of self‐reported eye disease and a visual acuity of 0.8 or above.

**Results:**

Analyses were done on data from 42 741–42 879 participants (considering only complete responses for each model) and showed that high refractive errors (≤ −6 D and ≥ +6 D) were significantly associated with diminished self‐reported visual function and visual concern. High myopia was associated with lower self‐perceived present eyesight (OR = 1.89, 95% CI: 1.69–2.11, *p* < 0.001), increased vision concerns (OR = 1.78, 95% CI: 1.59–1.99, *p* < 0.001), reduced self‐perceived accomplishment (OR = 1.14, 95% CI: 1.01–1.28, *p* < 0.05) and greater vision‐related limitations (OR = 1.44, 95% CI: 1.28–1.61, *p* < 0.001). High hypermetropia was similarly associated with lower self‐perceived present eyesight (OR = 4.23, 95% CI: 3.05–5.87, *p* < 0.001), increased vision concerns (OR = 2.15, 95% CI: 1.51–3.07, *p* < 0.001), reduced self‐perceived accomplishment (OR = 2.83, 95% CI: 1.99–4.01, *p* < 0.001) and greater vision‐related limitations (OR = 2.78, 95% CI: 1.94–3.97, *p* < 0.001).

**Conclusion:**

The present findings demonstrate a statistically significant negative correlation between high refractive errors and self‐reported visual function as well as decreased visual concern. They provide a new perspective on the impact of high refractive errors and highlight the importance of further research aimed at preventing their development.

## 1 INTRODUCTION

Refractive errors are optical imperfections that prevent the eye from properly focusing light on the fovea, causing blurred vision. They are categorized mainly into myopia, hypermetropia, astigmatism and presbyopia, each with distinctive attributes and implications (Harb & Wildsoet, 2019).

Globally, the prevalence of myopia is rising and projections estimate that by 2050 half of the global population will be myopic, with 10% manifesting high myopia (defined as ≤ −5.00 dioptre (D) by Holden et al., 2016). Unlike other prevalent ocular conditions, such as cataract and glaucoma, myopia typically manifests early, often during an individual's formative years, impacting a significant portion of an individual's life (Morgan & Rose, 2005). Studies indicate that an early onset of myopia increases the risk of progressing to high myopia (Hu et al., 2020). This in turn increases the risk of sight‐threatening conditions such as primary open‐angle glaucoma, retinal detachment, myopic maculopathy and choroidal neovascularization (Haarman et al., 2020). Not only does this impair patients' visual acuity but can also lead to increased anxiety and psychological stress (Yokoi et al., 2014). Similarly, hypermetropia carries a risk of developing conditions such as primary angle‐closure glaucoma and strabismus and can have a significant impact on a person's ability to perform close‐up tasks (Shen et al., 2016; Zhu et al., 2015).

Besides the obvious effect on visual acuity, both uncorrected myopia and hypermetropia have a socio‐economic impact. This includes expenses associated with glasses or contact lenses, opting out of attending physical spare time interests due to the issue of wearing spectacles as well as feeling socially limited as a result of the appearance with glasses (Berdahl et al., 2020; Chua & Foster, 2020; Goverdhan et al., 2011; Queirós et al., 2012; Yang et al., 2021). Refractive errors also have significant implications on the quality of life (Kandel et al., 2017; Sankaridurg et al., 2021; Zhu et al., 2021). The impairment of quality of life in some individuals with refractive errors is evident in everyday challenges such as reading subtitles on TV, recognizing people on the street and the ability to stay engaged during a presentation or lecture (Hays et al., 2003).

Refractive errors and correction hereof are typically evaluated through standard clinical measurements of visual acuity and refractive error such as retinoscopy, autorefraction and cycloplegic refraction measurement (Al Bahhawi et al., 2018; Congdon et al., 2008; Gifford et al., 2019). While these measurements provide necessary clinical information when choosing and evaluating the treatment of refractive error, they fail to evaluate the consequences of daily challenges or subjective well‐being. Patient‐reported outcome measures have previously been shown to be of great significance when predicting mortality (Miilunpalo et al., 1997) as well as assessing disease progression (Friis et al., 2021). Patient‐reported eye health has also shown an association with low quality of life and mental illness (Berdeaux et al., 2007; Lundeen et al., 2022; Wang et al., 2023). Therefore, it is relevant to take self‐reported visual function into consideration when evaluating treatment effects in patients with refractive errors. The purpose of correcting refractive errors should not merely be to improve visual acuity but to maximize the subjective well‐being and functionality of the individuals.

This study investigated the association between refractive errors and visual function, defined by a question regarding self‐perceived present eyesight and being limited in daily activities, in an eye‐healthy cohort. This study also studied the impact of refractive errors and visual concern, assessed through questions regarding concern about vision and a feeling of accomplishing less because of their vision. The outcomes of this study shed light on whether individuals with high refractive measures have greater concerns and limitations regarding their visual function. Thus adding another perspective to the implications of high refractive errors.

## 2 METHODS

This study is based on data collected as part of Project FOREVER (Finding Ophtalmic Risk and Evaluating the Value of Eye exams and their predictive Reliability), which is a Danish eye and vision cohort. The project received approval from the Danish National Committee on Health Research Ethics, with the project code H‐21026000. The design and methodology of Project FOREVER have been described in detail by Freiberg et al., 2024 and is briefly summarized here. In this study, we used the data and questionnaire responses from Project FOREVER.

### 2.1 Project FOREVER

Project FOREVER is a collaboration between researchers at the University of Copenhagen and the optician chain Synoptik A/S. Participants aged 18 and above were recruited from about 100 different Synoptik stores across Denmark, and all participants gave their informed written consent before inclusion.

Upon consent eye examination data from the optician store was obtained. Aside from eye examination data, Project FOREVER also features an online questionnaire. The validation stage of the questionnaire incorporated comprehensive interviews and gathered feedback from a varied group of 20 participants. Accessible through a web link on any device with internet capability, the questionnaire covers, among others, areas like ocular disorders, overall ocular and health conditions and psychological well‐being. It is comprised of 13 distinct sections and over 270 questions. Certain questions are stratified into primary, secondary or tertiary tiers, facilitating tailored questions based on prior answers. In specific instances, respondents can provide open‐ended responses to account for uncommon conditions.

In this study we looked at answers to specific questions from the FOREVER questionnaire regarding self‐perceived visual function and visual concern. We assessed participants' visual function and visual concern from four questions from the FOREVER questionnaire (Figure 1). These questions have either 5 or 6 possible answers with 1 being the best category and 5 or 6 being the poorest category in the first two questions and 1 being the poorest category and 5 being the best category in the two remaining questions.

**FIGURE 1:**
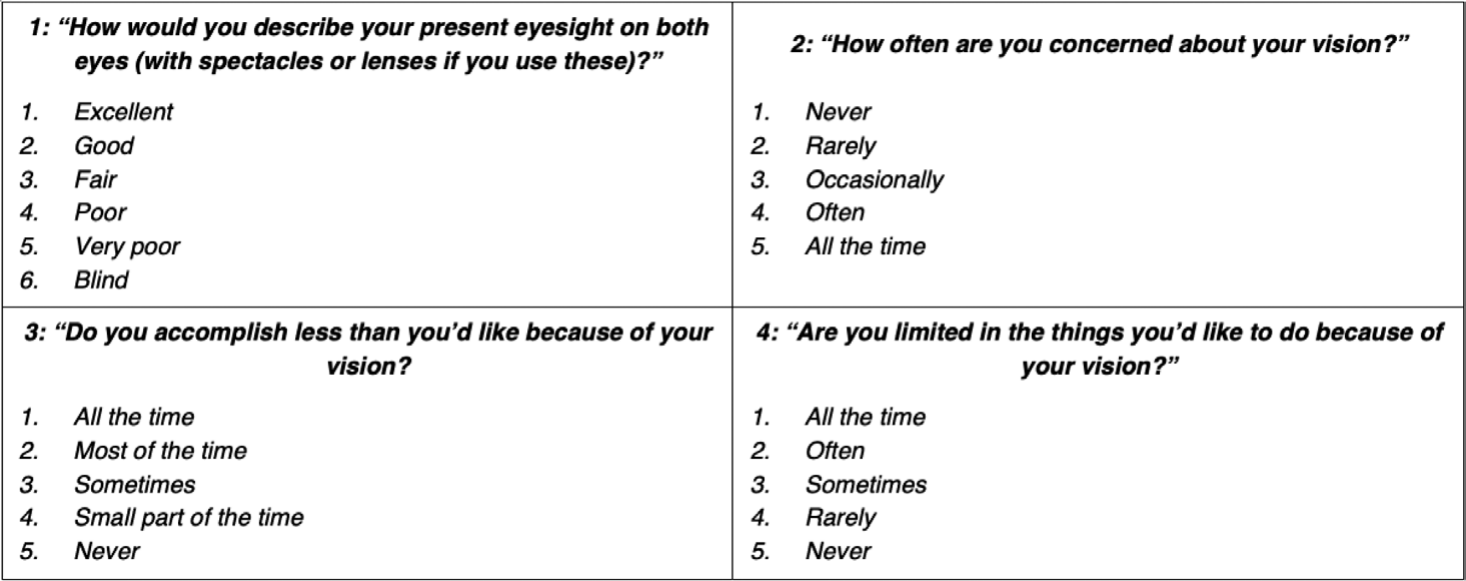
Questions regarding visual function and visual concern. The four different questions from the FOREVER questionnaire used to assess the visual function and visual concern of individuals with refractive errors.

The refractive errors and visual acuity were included in the eye examination data from the optician stores measured by qualified and autorefraction‐certified optometrists. Information on age, gender, eye disease and responses to the four questions were self‐reported and recorded using the FOREVER questionnaire.

### 2.2 Inclusion and exclusion criteria

Individuals with an available refractive measurement (myopia, hypermetropia, emmetropia or astigmatism) were included in this study. The measurements were based on non‐cycloplegic refractive errors. Participants who described their present eyesight as being blind (*n* = 3) and individuals with self‐reported eye diseases were excluded to ensure an eye‐healthy cohort (*n* = 8.055). Self‐reported eye diseases included glaucoma, cataract, age‐related macula degeneration, central serous chorioretinopathy, genetic retinal diseases (including retinitis pigmentosa and Stargardt disease), uveitis, retinal detachment, blood clot in the eye, posterior vitreous detachment, diabetic retinopathy, keratoconus, optic disc drusen and mole in one or both eyes. See appendix 1 for exact exclusion numbers.

Furthermore, participants without a recorded visual acuity or visual acuity of below 0.8 were excluded to ensure an eye‐healthy cohort. Visual acuity of 0.8 and above was chosen, as this cut‐off has been applied by several other studies (Bergman & Sjöstrand, 2002; Dawes et al., 2014; Laitinen et al., 2005).

### 2.3 Data and statistics

To maintain data integrity, we exclusively considered complete responses, omitting any incomplete responses. As a result, the datasets for each model differed slightly in size (42741–42 879 participants). We excluded participants with a refractive error below −15.0 D or above +15.0 D for refractive measures, to minimize the influence of extreme outliers. The analyses were carried out in R, version 4.4.0 (R Core Team, 2022).

To investigate associations we used logistic regression (Modern Applied Statistics With S, 2004) with weights based on class frequency to account for the large imbalance in responses (see Table 2 for distribution of responses). In order to further investigate if the distribution of our data matched an expected theoretical distribution, we did a Chi‐Square Goodness of Fit Test on the regression model for each question. ANOVA tests were done on the covariates to see if they contributed to the model. To counteract for multiple comparison Holm's correction was used.

When defining the refraction, we calculated the spherical equivalent (SER) (cylinder/2 + sphere). In our models, we used the SER derived from the right eye. After determining the SER, we grouped the cohort into intervals depending on the dioptre (D), which can be found in Figure 2. The intervals for myopia were based upon a proposed set of standards for myopia classifications for epidemiologic studies from Flitcroft et al., 2019. The same classification and intervals were mirrored for hypermetropia. We defined high refractive error as ≤ −6 D and ≥ +6 D.

**FIGURE 2:**
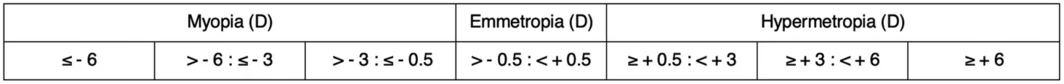
Division of intervals of refractive measures. Measured in dioptre.

We used refractive errors between > −0.5: < +0.5 as the emmetropic reference group in the regression analyses. The models investigating the association between the different questions and refractive errors were adjusted for sex and age.

## 3 RESULTS

### 3.1 Demographics

The age range was 18 to 93 years. The overall mean age was 53.3 years (SD ±14.2 years), and the overall sex distribution showed a majority of females (61.7%). A predominance of females was seen across all refractive intervals. The dataset included a total of 16 421 male and 26 458 female participants. Details of the distribution of the characteristics across the various refractive intervals can be found in Table 1. The four datasets contained 42 879 observations (Present eyesight), 42 741 observations (Concerns about vision), 42 748 observations (Accomplishing less) and 42 748 observations (Vision‐related limitations).

**TABLE 1:** Distribution of age, sex and total count.

SER (dioptres)	Mean age (SD)	Sex (M/F)	Total count
≤ −6	48.1 (±12.8)	381/787 (32.6%/67.4%)	1168
> −6: ≤ −3	49.4 (±13.8)	1693/2773 (37.9%/62.1%)	4466
> −3: ≤ −0.5	49.4 (±15.2)	4496/7331 (38%/62%)	11 827
> −0.5: < +0.5	51.6 (±13.6)	4514/7413 (37.8%/62.2%)	11 927
≥ +0.5: < +3	60.1 (±11.1)	4722/7194 (39.6%/60.4%)	11 916
≥ +3: < +6	60.0 (±13.2)	570/905 (38.6%/61.4%)	1475
≥ +6	57.8 (±13.1)	45/55 (45%/55%)	100
Total	53.3 (±14.2)	16 421/26458 (38.3/61.7%)	42 879

*Note*: Mean age (and standard deviation), sex distribution (and percentage) and total count across the different refraction intervals.

Generally, the distribution of refractive measurements appeared more centred around the emmetropic values, which is visualized in Figure 3. The majority of the total cohort fell in the intervals of [> −0.5: ≤ +0.5], [≥ +0.5: < +3] and [> −3: ≤ −0.5]. The dataset contained 17 461 myopic participants ([≤ −6: ≤ −0.5]) but only 13 491 hypermetopic participants ([≥ +0.5: ≥ +6]). 11 927 individuals of the cohort were defined as emmetropic in the refractive interval of [> −0.5: < +0.5].

**FIGURE 3:**
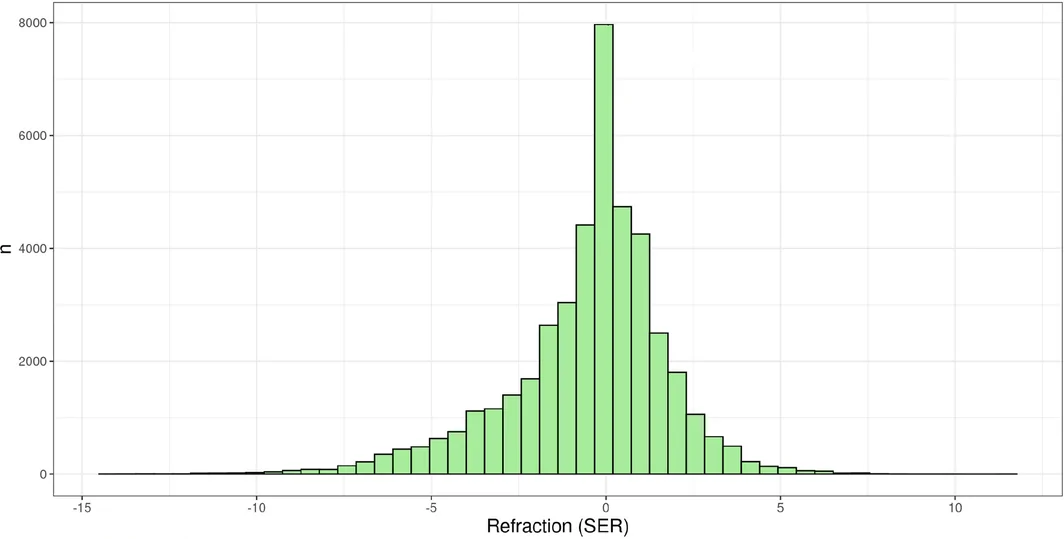
Distribution of refractive errors. Distribution of refractions of the right eyes in the FOREVER cohort. Spherical equivalent (SER).

### 3.2 Distribution of responses

Regarding the first question, assessing their present eyesight, the majority of the responders chose either rating 2 (Good) (59.6%) or rating 3 (Fair) (25.2%). In the second question, assessing how much the responders concern about their eyesight, most of them chose either rating 2 (Rarely) (41.6%) or rating 3 (Occasionally) (39.9%). For the remaining two questions, assessing, respectively, if they accomplish less because of their sight and if they are limited because of their vision, most responders chose to rate 5 (Never) (74.2% and 57.3%, respectively). The total distribution of the answers to the questions can be found in Table 2.

**TABLE 2:** Distribution of responses.

Rating	Present eyesight	Concerns about vision	Accomplishing less	Vision‐related limitations
	*n* (%)	*n* (%)	*n* (%)	*n* (%)
1	5451 (12.7%)	4725 (11.1%)	69 (0.2%)	83 (0.2%)
2	25 567 (59.6%)	17 783 (41.6%)	262 (0.6%)	553 (1.3%)
3	10 790 (25.2%)	17 074 (39.9%)	2492 (5.8%)	4107 (9.6%)
4	1017 (2.4%)	2887 (6.8%)	8219 (19.2%)	13 515 (31.6%)
5	54 (0.1%)	272 (0.6%)	31 706 (74.2%)	24 490 (57.3%)
Total	42 879	42 741	42 748	42 748

*Note*: Distribution of responses to the four questions from the FOREVER questionnaire assessing self‐reported visual function and visual concern. ‘Present eyesight’ rating 1: ‘Excellent’, rating 2: ‘Good’, rating 3: ‘Fair’, rating 4: ‘Poor’, rating 5: ‘Very poor’. ‘Concerns about vision’ rating 1: ‘Never’, rating 2: ‘Rarely’, rating 3: ‘Occasionally’, rating 4: ‘Often’, rating 5: ‘All the time’. ‘Accomplishing less’ rating 1: ‘All the time’, rating 2: ‘Most of the time’, rating 3: ‘Sometimes’, rating 4: ‘Small part of the time’, rating 5: ‘Never’. ‘Vision‐related limitations’ rating 1: ‘All the time’, rating 2: ‘Often’, rating 3: ‘Sometimes’, rating 4: ‘Rarely’, rating 5: ‘Never’.

The answers to each question where dichotomized by dividing them into a ‘positive’ or ‘negative’ answer. Rating 1 to 3 from the first two questions (Present eyesight and Concern about vision) were categorized as ‘positive’ and rating 4 and 5 as ‘negative’. Ratings 1 to 3 from the third and fourth questions (Accomplished less and Vision‐related limitations) were categorized as ‘negative’ and rating 4 and 5 as ‘positive’. Table 3 showed the dichotomized distribution of answers.

**TABLE 3:** Dichotomized distribution of responses.

Rating	Present eyesight	Concerns about vision	Accomplishing less	Vision‐related limitations
	*n* (%)	*n* (%)	*n* (%)	*n* (%)
Positive	41 808 (97.5%)	39 582 (92.6%)	39 925 (93.4%)	38 005 (88.9%)
Negative	1071 (2.5%)	3159 (7.4%)	2823 (6.6%)	4743 (11.1%)

*Note*: Distribution of responses to the four included questions from the FOREVER questionnaire assessing self‐reported visual function and visual concern categorized into ‘positive’ and ‘negative’ ratings.

The overall tendency was that high refractive errors showed higher odds of rating negative compared to the reference for all of the four logistic regression models. That being for both myopia and hypermetropia, which is illustrated in Figure 4.

**FIGURE 4:**
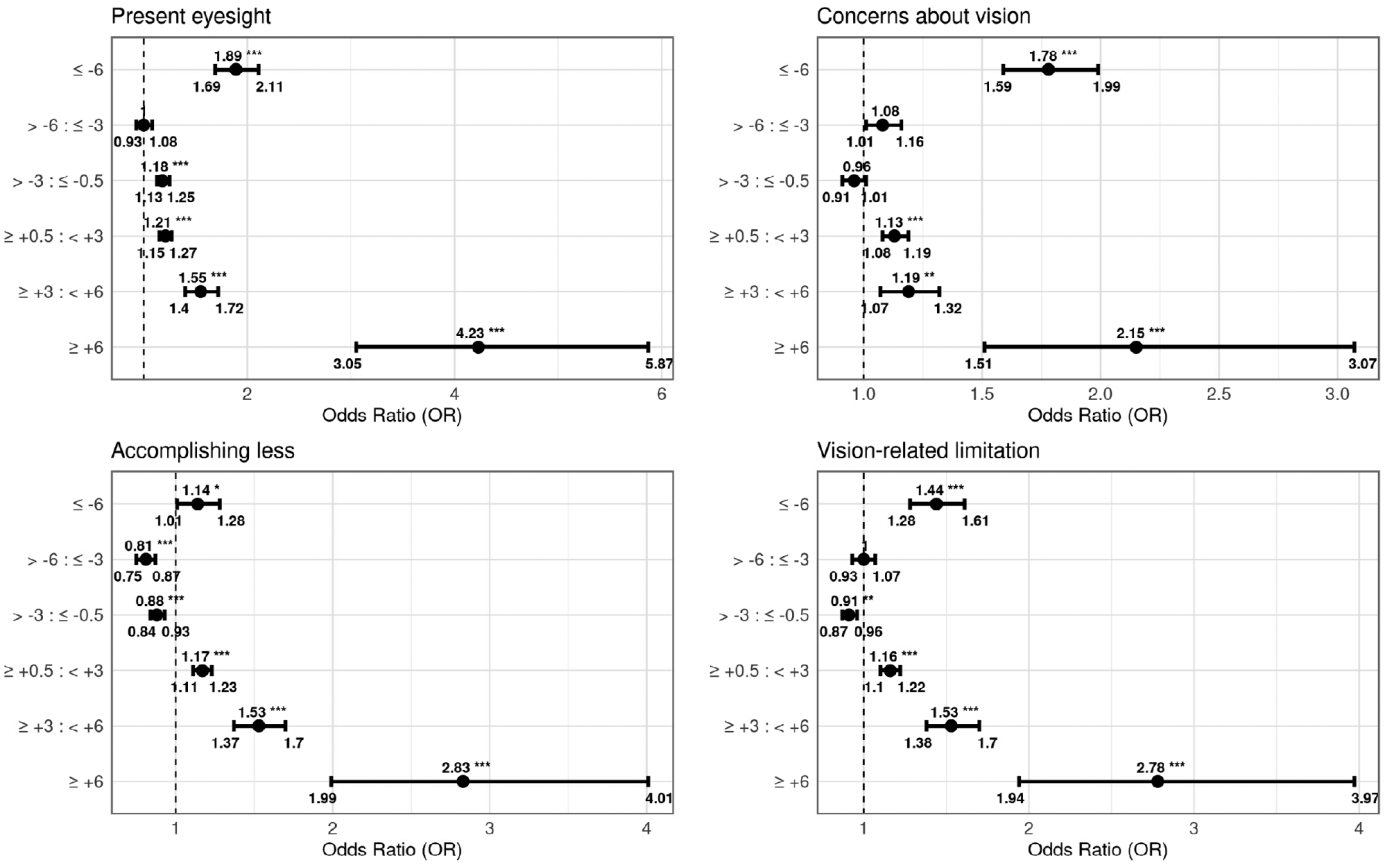
Association between refractive errors and self‐reported visual function and visual concern. Odds ratios (OR), 95% confidence intervals (CI) and adjusted p‐values (multiple testing) for the four different models assessing the association between self‐reported visual function and refractive errors (as SER), as well as self‐reported visual concern and refractive errors (as SER) in the FOREVER cohort. The reference group where emmetropic. *p < 0.05, **p < 0.01, ***p < 0.001.

In the first model (Present eyesight), the odds of falling into the negative category increased with each subsequent interval of refractive error when comparing to reference, apart from [> −6: ≤ −3], with the odds increasing more for hypermetropic eyes.

In the second model (Concerns about vision), the odds of falling into the negative category also increase with each subsequent interval of refractive error when comparing to reference for the hypermetropic eyes and high myopic eyes. [> −3: ≤ −0.5] and [> −6: ≤ −3] showed no statistically significant increase.

For the third model (Accomplishing less), we found that the odds of falling into a poorer category increased with each subsequent interval of refractive error when comparing to the reference for the hypermetropic and high myopia eyes. For [> −3: ≤ −0.5] and [> −6: ≤ −3] the odds, interestingly, decrease slightly.

For the fourth model (Vision‐related limitations), we similarly found that the odds of falling into a poorer category increased with each subsequent interval of refractive error when comparing to reference emmetropia for the hypermetropic and high myopia eyes, apart from the group [> −6: ≤ −3]. For the group [> −3: ≤ −0.5], the odds decrease slightly. The odds ratios increase more in hypermetropic eyes in this model.

An overview of the odds ratios depicting the association between the different refractive intervals and the four questions from the FOREVER questionnaire can be seen in Table 4 and Figure 4.

**TABLE 4:** Association between refractive errors and self‐reported visual function and visual concern.

SER (dioptres)	Present eyesight		Concerns about vision		Accomplishing less		Vision‐related limitations	
	OR	CI (95%)	OR	CI (95%)	OR	CI (95%)	OR	CI (95%)
≤ −6	1.89***	1.69–2.11	1.78***	1.59–1.99	1.14*	1.01–1.28	1.44***	1.28–1.61
> −6: ≤ −3	1.00	0.93–1.08	1.08	1.01–1.16	0.81***	0.75–0.87	1.00	0.93–1.07
> −3: ≤ −0.5	1.18***	1.13–1.25	0.96	0.91–1.01	0.88***	0.84–0.93	0.91**	0.87–0.96
≥ +0.5: < +3	1.21***	1.15–1.27	1.13***	1.08–1.19	1.17***	1.11–1.23	1.16***	1.10–1.22
≥ +3: < +6	1.55***	1.40–1.72	1.19**	1.07–1.32	1.53***	1.37–1.70	1.53***	1.38–1.70
≥ +6	4.23***	3.05–5.87	2.15***	1.51–3.07	2.83***	1.99–4.01	2.78***	1.94–3.97
*n*	42 879		42 741		42 748		42 748	
Goodness of fit *p*‐value	0.483		0.486		0.458		0.485	

*Note*: Overview of the odds ratios (OR), 95% confidence intervals (CI) and adjusted *p*‐values (multiple testing) for the different spherical equivalents (SER) (in dioptres) for the questions of self‐reported visual function and visual concern in the FOREVER questionnaire as well as *p*‐values for the Pearson's Goodness of Fit Test for each model.

*p < 0.05; **p < 0.01; ***p < 0.001.

## 4 DISCUSSION

Our study on self‐reported visual function and visual concern showed that individuals with refractive errors, particularly high myopia and high hypermetropia, have an increased odds ratio of responding negatively to all four questions compared with the emmetropic reference. This association indicates that individuals with high refractive errors experience a negative impact on their daily lives. These results are in line with previous research that has investigated the impact of refractive errors on socio‐economics and quality of life (Berdahl et al., 2020; Chua & Foster, 2020; Goverdhan et al., 2011; Sankaridurg et al., 2021). While our results focus more on visual function and visual concern, other studies have focused on other aspects of the effects of refractive errors. As an example, a review on myopia found that high myopes in particular often experience decreased quality of life because of psychological, cosmetic, practical and financial impacts, which affect productivity, mobility and daily activities (Chua & Foster, 2020). Similarly, a review of the burden of presbyopia found that presbyopia could affect mental well‐being, with 15% of patients feeling that they accomplished less and 7% feeling embarrassed due to their visual impairment (Berdahl et al., 2020).

A large proportion of respondents, especially those with high refractive errors, reported concerns about their vision. The increased vision‐related concern is in accordance with what has been documented in a qualitative study by Kandel et al., 2017, highlighting the impact of myopia on the quality of life. The study was conducted qualitatively comprising of interviews with 48 individuals with refractive error. This was in contrast to our quantitative study utilizing a questionnaire across a much larger cohort. Moreover, the study supported the notion that individuals with high refractive errors often felt restricted in their daily activities due to their vision, further underlining previous concerns about the broader socio‐economic implications of these visual conditions.

A quantitative study using a questionnaire by Pesudovs et al. (2006) investigated how refractive surgery improved the quality of life in people with refractive errors. They similarly found that quality of life was lowest in spectacle wearers, particularly those with high refractive errors. Several studies have found a positive effect of refractive surgery on quality of life (McDonnell et al., 2003; Schein, 2000). This is consistent with our findings of negative self‐reported visual function and visual concern in individuals with high refractive errors. Refractive surgery often leads to increased independence in daily activities, enhancing both professional and personal experiences.

One of the major strengths of this study lies in its use of the FOREVER questionnaire, a comprehensive tool that incorporates a wide spectrum of aspects related to visual and general health. Additionally, the large number of observations in our cohort supports the reliability of our findings from our analyses. Additionally Synoptik A/S does not cater specifically to only low‐ or high‐income clientel. They attract a broad consumer base reflective of the larger Danish population.

However, while this study utilizes data of self‐reported nature, inherent biases in self‐reported outcomes cannot be disregarded. Subjectivity in responses could be influenced by various factors unrelated to the refractive error itself. Particular attention should be given to the potential introduction of social desirability bias where there is a tendency of respondents to answer questions in a manner that will be viewed favourably by others and response shift bias where individuals change their internal standards, values or conceptualization of a concept, leading to changes in their self‐assessment over time (Bergen & Labonté, 2020; Bispo Júnior, 2022; Latkin et al., 2016). This should especially be considered given the chronic nature of refractive errors when untreated. The presence of central tendency bias, where tendency of respondents to avoid extreme responses, could further influence the accuracy of the results (Allred et al., 2016; Douven, 2018).

The cross‐sectional nature of our study also limits the possibility of establishing potential causal relationships, and a longitudinal study design might have provided a more dynamic view of how self‐perceived visual function and visual concern evolves in relation to refractive error. While our study only distinguished strictly between refractive errors defined as myopia or hypermetropia based on SER, future research should differentiate further between refractive errors such as astigmatism, presbyopia and anisometropia, hereby creating a clearer picture of how the different types of refractive errors uniquely affect people's well‐being. Since the refractive measurements were based on non‐cycloplegic refractive errors, it may likely provide accurate results in adults, but in younger participants, refractive error may be slightly underestimated due to latent hyperopia and pseudomyopia.

This study has a majority of female participants, which possibly affects the results of this study. It is well documented that females are more likely to participate in research compared to men (Tolonen et al., 2015). The gender imbalance brings some uncertainty to how well the studied population reflects the general Danish population, which is close to 50% male and 50% female (Statistics Denmark).

While our data provides insights across a spectrum of refractive errors, the cohort representing more pronounced hypermetropia was notably smaller (*n* = 100), resulting in a rather wide confidence interval. Therefore, the estimates are associated with greater uncertainty, which might affect the precision and reliability of the conclusions drawn from this subgroup. It's important to consider this when making interpretations or generalizations based on these findings. It is consistent with epidemiological data that the sample size for high hypermetropia is smaller relative to other refractive categories, given the lower prevalence of high hypermetropia in the general population (Vitale, 2008; Williams et al., 2015).

Our findings demonstrating the negative impact of increasing refractive error on self‐reported visual function and visual concern underlining the importance of addressing both preventative and interventional approaches to high refractive errors. Preventive strategies especially for myopia already exist (Jonas et al., 2021; Yam et al., 2022). However, modalities that limit the development and progression of significant refractive anomalies should be further studied. Furthermore, for those already affected, providing access to spectacles, contact lenses or refractive surgical interventions becomes critical and should be considered. It's noteworthy that Denmark has taken a proactive step in this direction. Unique in its approach on a global scale, Denmark offers refractive surgery within the Danish public healthcare system specifically for those presenting with high refractive errors defined as ≤ −6 D and ≥ +6 D or anisometropia or astigmatism of ≥3 D in at least one eye (Dansk Oftalmologisk Selskab (DOS), 2020). Such initiatives not only underscore the significance of addressing visual impairment but also highlight the potential for other nations to adopt similar strategies. It is, however, important to underscore that the future solution to high refractive errors is not refractive surgery, but the prevention of the development of high refractive errors, due to the fact that surgery in itself is not without risks of complications (Lee et al., 2016). Furthermore, surgery does not diminish the inherent risk of complications related to high refractive errors.

## 5 CONCLUSION

In conclusion, our study found that participants with high refractive errors assessed their vision worse, were more concerned about their vision, had feelings of having accomplished less and felt limited in everyday life compared with emmetropic participants. We found high odds of reporting negatively with increasing refractive error for hypermetropia and high myopia. While clinical measurements such as visual acuity and refractive error measurement remain pivotal, integrating patient perspectives offers the potential for a more comprehensive and empathetic approach to future eye care. This further highlights the need for more preventive strategies or reduction in refractive errors to improve self‐perceived visual function and well‐being, and potentially reduce the risk of long‐term complications related to high refractive errors.

## CONFLICT OF INTEREST STATEMENT

Jens Riis Møller, Arevak Saruhanian, Josefine Freiberg, Marcel Reimann, Jeppe Nygård Samuelsen, Miriam Kolko and Nina Jacobsen have no conflict of interest in relation to this study. Jens Rovelt is currently an employee of Astellas Pharma Denmark A/S.

## APPENDIX A

### A.1 Number of participants excluded by eye disease

**Table:**

Eye disease	Numbers excluded (n)
Glaucoma	945
Cataract	3252
Age‐related macula degeneration	749
Centrel serous chorioretinopathy	70
Genetic reinal diseases (including retinitis pigmentosa and Stargardt disease)	72
Uveitis	251
Retinal detachment	345
Blood clot in the eye	200
Posterior vitreous detachment	762
Diabetic retinopathy	129
Keratoconus	63
Optic disc drusen	36
Mole in one or both eyes	194
Malignant melanoma in the eye	11
Other eye disease	976
Total count	8.055

*Note*: Distribution of self‐reported eye diseases excluded from this study.
